# Ring Chromosome 18: A Case Report

**Published:** 2014

**Authors:** Shermineh Heydari, Fahimeh Hassanzadeh, Mohammad Hassanzadeh Nazarabadi

**Affiliations:** Department of Medical Genetics, Faculty of Medicine, Mashhad University of Medical Sciences, Mashhad, Iran.

**Keywords:** Ring chromosome 18, karyotyping, mental retardation

## Abstract

Ring chromosomes are rare chromosomal disorders that usually appear to occur de novo. A ring chromosome forms when due to deletion both ends of chromosome fuse with each other. Depending on the amount of chromosomal deletion, the clinical manifestations may be different. So, ring 18 syndrome is characterized by severe mental growth retardation as well as microcephaly, brain and ocular malformations, hypotonia and other skeletal abnormalities. Here we report a 2.5 years old patient with a cleft lip, club foot, mental retardation and cryptorchidism. Chromosomal analysis on the basis of G-banding technique was performed following patient referral to the cytogenetic laboratory. Chromosomal investigation appeared as 46, XY, r(18) (p11.32 q21.32). According to the clinical features of such patients, chromosome investigation is strongly recommended.

Ring chromosomes from the cytogenetic point of view are rare forms of chromosomal structure abnormalities (1, 2). The ring chromo-some has been reported for all human chromosomes; their frequency is estimated to be between 1/30000 to 1/60000 (3) and almost 50 percent of all ring chromosomes originate from acrocentric chromosomes (2). Ring chromosomes often occur de novo (-) and the incidence of inherited cases is 1 percent. On the other hand, 90 percent of these cases are of maternal origin; since ring chromosome will cause spermatogenesis abnormalities and consequently infertility in affected men (1, 2).

The classic form of ring chromosome formation is the occurrence of a break in both arms and fusion of the breaking points together which result in the loss of terminal segments and genetic material. This rearrangement causes partial monosomy of the distal region of both arms (3, 5). Up to 2011 only 70 cases of ring chromosomes have been reported, in which among them, ring chromosome 18 was almost the most common (3, 5, 6).

The phenotypic features of the ring chromo-some 18 patients are similar to the features of the 18 q deletion syndrome patients and a few of them are similar to 18 p deletion or the combination of these two syndromes (4, 7). On the other hand, patients with ring chromosomes often exhibit a general overlap in clinical manifestations and depending on the size and the amount of the euchromatin loss, the phenotype is variable (1, 2, 4, 8). Therefore, the intensity of the phenotype features depends on the size of chromosome deletion, the stability of the rings, the presence of monosomy and other secondary aneuploidy cell lines, and the occurrence of mosaicism (8).

The clinical manifestations of ring chromo-some 18 patients are include microcephaly, mild to severe mental retardation, short stature, obesity, mi-cropenis, cryptorchidism, hypertelorism, epicanthic fold, microgenathia and small hands (7). Other manifestations include hypotonia, foot deformation, proximally placed thumbs,  arthritis/stenotic ear canals, long tapering digits, abnormal male genita-lia, flat midface, prominent antihelix antitragus and carp - shaped mouth (4) as well as immunological problems (3, 6).

## Case Report

Here we report a patient who is 2.5 years old with a cleft lip, club foot, mild mental retardation and have some changes in the genital region and the testicles may not be fully descended (cryptor-chidism). He has punctured eardrum and uses hearing aids.

Chromosomal analysis was performed accor-ding to standard procedures using GTG-banding. Peripheral blood lymphocytes were cultured in RPMI 1640 medium (Gibco®) enriched with FBS, phytohemagglutinin and L-glutamine. The cells were cultured for 72 hours at 37 °C in a CO2 incubator. Cultures were stopped by adding colcemid solution 2 hours before harvesting, then the cells were exposed to hypotonic solution (KCl 0.075 M); fixed with methanol/ acetic acid (3:1) (Vol/Vol). Metaphase chromosome spread was prepared and G- banding technique was applied with the use of trypsin-giemsa (GTG). A minimum of 50 metaphases were examined and all showed ring chromosome 18. Karyotypes were assigned according to the International System of Human Cytogenetic Nomenclature (ISCN) 2005.

**Fig. 1:**
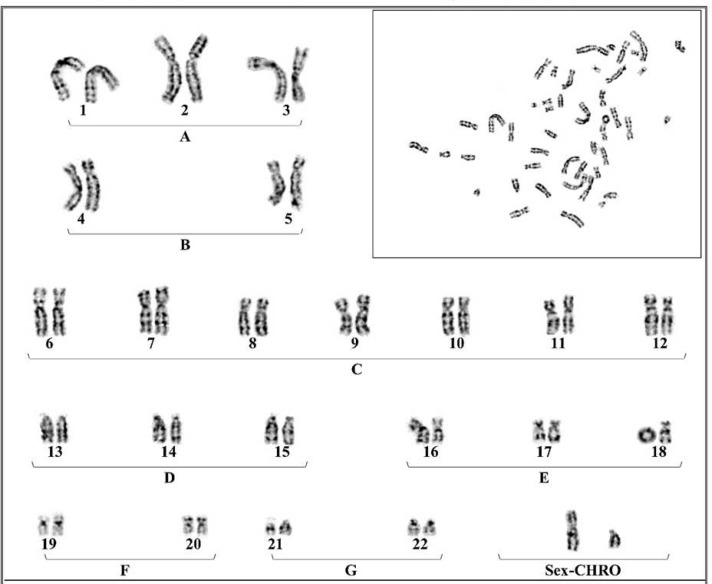
2.5 years boy with ring chromosome 18. 46, XY, r (18) (p11.32 q21.32).

Chromosome analysis appeared as 46, XY, r(18) (p11.32 q21.32) (fig.1) whereas the parents karyotype appeared normal (46, XX and 46, XY). 

Therefore, this abnormality seems to have appeared de novo.

## Discussion

Ring chromosomes 18 form when both ends of the chromosome break and the broken sticky ends fuse at the breakage point. During this process, some of the genetic materials may be lost and cause the clinical manifestations. GTG banding karyotype in our patient revealed that the 18p11.32 and 18q21.32 regions were lost. Previous reports have presented ring chromosome 18 as a rare and mostly appearing in mosaic form (1, 7). So far, one case of mosaic ring chromosome 18 has been reported in Iran (9). Our patient revealed a phenotype similar with other reported cases (7, 10, 11), but the most interesting finding which differenciates our finding with other investigations is the deletion of 18q21.33 region. Databases such as NCBI and OMIM (*159430) show that an important gene called MBP (myelin basic protein) is located at this region. This gene encodes a protein which is incorporated in oligodendrocytes and schwann cells myelin sheet (12). The correlation between the severe degrees of mental retardation in patients with deletions proximal to 18q21.31, and milder forms in patients with deletions distal to 18q21.33 was reported (6).This finding is comparable with the result of Netzel et al.  (13). Thus, in our patient, mental retardation may be related to the deletion of both 18q21.32 region and MBP gene. Our finding also demonstrate that in such cases, despite normal parents' karyotype, chromosomal investigation is strongly recommended and may be informative as well as other molecular cytogenetic techniques such as FISH and CGH array which can help to achieve a better understanding of the mechanism of ring chromosome 18 formation and clinical mani-festations.
